# Observations on Dr. Pearson's Evidence

**Published:** 1802-09-01

**Authors:** 


### To Dr. BRADLEY.

Sir,

The last Number of your Journal, has, I am persuaded, afforded sincere pleasure to every friend of improvement and humanity, humanity, by announcing the final decifion of Parliament on the important fubject of Vaccine Inoculation. With others, I muft regret the very inadequate reward conferred on Dr. Jenner, for one of the greateft difcoveries on the records of medical fcience; but as this feeling is general, and has fuggefted the idea of an univerfal contribution, I have no doubt but the patriotifm and liberality of the country will be roufed, and that our national gratitude will be vindicated by a fubfcription commenfurate to the benefit which Dr. J. has conferred on fociety. In perufing the Reports of the Committee of the Houfe of Commons, I have, with many others, been ftruck by the evidence of Dr. Pearfon, as a very extraordinary attempt to diminifh the well-earned reputation of Dr. Jenner, which has been exhibited to a difcerning public. Although I have obferved that Dr. Jenner (knowing the folid foundation on which his reputation refts) has conftantly difregarded the attempts of Dr. Pearfon to injure him; yet it is impoffible for his friends any longer to remain filent lookers-on. I fhall therefore make fome Remarks on the evidence alluded to, in which perfonal animofity can have no fhare, as my acquaintance with Dr. Pearfon is derived folely from his writings. Impelled by refpect for the character of Dr. Jenner, and convinced that gratitude to the real benefactors of mankind is but an act of juftice, I think it becomes on the prefent occafion fuch an act to animadvert with freedom on the conduct of Dr. P.

You have, Sir, very truly remarked, that all Difcoveries have been oppofed on the ground of originality. On this fubject, I remember to have heard it well obferved, that all difcoveries of effential benefits to mankind were uniformly attacked in three fucceffive ways. Firft, the reality of the difcovery is denied. Succeeding experiments corroborate its truth, and then we are told, that although true it is of little value. Farther inveftigation refutes this affertion; and in the laft place, we are informed that the difcovery is not recent, but to be found in the mufty pages of fome author of antient date. The Hiftory of Science proves the juftice of this remark, and the prefent inftance is an additional corroboration of its truth; for in each of thefe three ways has the difcovery of Dr. Jenner been attacked.

Although the fubject is an unpleafant one, it becomes neceffary to revert to the indelicacy of Dr. Pearfon's conduct to Dr. Jenner, from the firft moment in which this great difcovery was announced. No fooner was Dr. Jenner's work from the prefs, than we find Dr. Pearfon, although at that time totally ignorant of Vaccine Inoculation, publifhing a pamphlet, and inviting practitioners to correfpond with *him* on the fubject, with with no other apparent view, than to attract the public attention to his name, and bury that of the modeft difcoverer in oblivion. In the next place, we fee him bufied in inftituting a Difpenfary for Vaccine Inoculation. Here we might expect to find the name of Dr. Jenner occupy a diftinguifhed place; but to the aftonifhment of all, it was not once mentioned. So ftrongly impreffed was his Royal Highnefs the Duke of York with the impropriety of Dr. Pearfon's conduct in the whole of this affair, that he indignantly commanded his name, with which he had hitherto honoured the Inftitution as Patron, to be withdrawn. Had Dr. Jenner confented afterwards to occupy his proper place in the Inftitution, the name and fupport of his Royal Highnefs would ftill have been continued; but feeling as he ought to have done the indelicacy with which he had been treated, he very properly declined the honour. Such was the behaviour of Dr. P. at the commencement of the Inquiry, and his fubfequent conduct before the Committee, abundantly evinces, that time had not improved his ideas of delicacy or juftice. Let us now, Sir, take a fhort view of his evidence, the whole tendency of which is obvioufly to deprefs the claims of Dr. Jenner to parliamentary remuneration. His attack is conducted in a two-fold way. Firft, he endeavours to fhew, that Vaccine Inoculation was known long before the period on which Dr. J. announced his difcovery; and, fecondly, his own great exertions are brought forward, to imprefs the Committee with the idea of his claim for reward being equal.

With refpect to the firft, it is very extraordinary that Dr. Pearfon fhould imagine that all the proofs *he* could poffibly bring forward would have much weight with the Committee, for on the flighteft examination their futility becomes evident. His motives are beft known to himfelf; but a regard to truth forces us to believe, that they proceeded from a difpofition of mind rather uncandid. In his examination on the 14th, the authority of Dr. Heberden is made ufe of, to prove what on that gentleman's examination was found completely erroneous, for he could not fpeak to what Dr. P. had afferted he could; and of Mr. Battifcombe, he had no knowledge. On this it is unneceffary to make any comment, the conclufion muft be obvious. The next day Dr. Pearfon refolved to pufh his oppofition farther; and a bundle of papers was produced, to prove the plagiarifm of Dr. Jenner. But how, Sir, is this made out? The papers delivered by Mr. Keate, were written after Dr. Jenner had publicly talked of his difcovery; and it even appears from their contents, that the writer had no decifive ideas on the fubject. Such was the mighty mafs of evidence intended to crufh irrefragable facts now firmly eftablifhed. The result refult may be eafily conceived, Dr. Pearfon and his papers were treated with the *attention* they merited. In the whole of this proceeding Dr. P. feems not to have been aware of the unremitting exertions of the Committee, for had he imagined that his unqualified aflertions would have been put to the proof, he furely would have been cautious in hazarding them.

I come now to the comparifon made by Dr. Pearfon between *his* efforts and thofe of Dr. Jenner. Dr. P. afferts, that although Dr. J. fet on foot the inquiry, the practice was almoft entirely eftablifhed by other practitioners; and that the *collateral* facts which Dr. J. advanced have been completely difproved. One would imagine, Mr. Editor, from this, that Dr. J. had merely propofed an ingenious hypothefis, which was to ftand or fall by the experiments of others, and not by any of his own. But how different is the fact! After many years devoted to the fubject, and after repeated experiments made to convince himfelf of the fafety and efficacy of Vaccine Inoculation, Dr. Jenner at length announced his difcovery to the world with that confidence which ample experience had infpired, and accompanied it with a number of cafes to prove its truth. The cafe in general ftands thus: In confequence of experiments, truth is difcovered, and publifhed to the world; thefe experiments are repeated by others, and found accurate; furely then it requires no great fagacity to difcern, that the eftablifhment of the fact is due to the original difcoverer, and not to thofe who, for their own fatisfaction, repeat his trials, and find them juft. So it was in the prefent inftance, Dr. Jenner publifhed what experience had fully proved to him, and *succeeding* experimentalifts found his affertions to be correct.

Dr. Pearfon makes a great parade of the *new* facts which he had difcovered; but in the procefs of his examination, he is ftripped of his borrowed plumes. "He had already learnt that young infants might be inoculated with fafety;" and from whence did he learn? Why, from the very work to which he was indebted for all his knowledge on the fubject, from Dr. Jenner's firft publication, in which the cafe of his own infant is fully related. So much for the *new* fact; and, to the difgrace of the times, we have many of the fame ftamp. Fearlefs of affertion, Dr. P. boldly ftates, that he has overturned all the collateral facts publifhed by Dr. Jenner: Yet, in oppofition to this, every one of them, fingular to tell, (and which confers the higheft praife on Dr. J.) remains unfhaken. The origin of the difeafe from the horfe, at firft created fufpicion; but Dr. J's own experiments, fince fully confirmed by thofe of Doctor Loy, have completely eftablifhed its truth. So much, Sir, for Dr. Pearfon's facts. It is painful to criticife the fubfequent part of his examination, for it exhibits fuch evafion, as is little confiftent with a candid did mind in fearch of improvement. "Did you never hear of inoculation having been performed by Mr. Cline with matter furnifhed by Dr. Jenner, previous to the commencement of your Vaccine Inoculation? Such was the queftion; and what the anfwer? *Cannot diftinctly recollect!* Shameful evafion refpecting a fimple fact. But the caufe is obvious, for at the time this inoculation took place by Mr. Cline, Dr. Pearfon had afferted, that Dr. Jenner was not in poffeffion of any Vaccine matter. I fhall fpare Dr. P. and not pufh this anfwer any farther.

The laft part of his examination I fhall notice is his anfwer to the queftion refpecting *his* matter producing eruptions. "Was not the matter found fault with? "No, but people were *difappointed.*" Now, Sir, I muft contend, that between finding fault and being difappointed in fuch a cafe, there was no room to attempt drawing a diftinction, for the one muft have inevitably followed the other. In confequence of the matter fent by Dr. Pearfon, a difeafe pretty fimilar to fmall-pox was produced, which muft, of courfe, have occafioned very great difappointment; and as it was known that the matter fent by Dr. Jenner *never did occasion puftules*, *great fault* muft have been found with that *fent by Dr. P.* So great mifchief attended the matter fent by this Gentleman, that Vaccine Inoculation was in danger of falling into difrepute; and thus, in confequence of the *exertions* of Dr. P. there was reafon to fear that one of the greateft improvements of modern times would have been loft. But the perfevering induftry of Dr. Jenner and his friends overcame this unlooked for obftacle; and, contrary to the affertion of Dr. P. it is now afcertained beyond difpute, that Small-pox-like eruptions from genuine Vaccine matter cannot appear. Another important error Dr. P. has fallen into, is of very ferious confequence. He has ftated, that it is indifferent at what period of the puftule the matter is taken for fubfequent inoculation. To thofe, however, who wifh well to this valuable difcovery, and who have no perfonal views to ferve, it is well known that it is of the firft moment to attend to the period on which the matter is taken, and that from neglecting this caution a vaft number of errors have been committed and prejudice has been created againft Jennerian Inoculation. From this curfory review of Dr. Pearfon's evidence, I think we may pretty fairly appreciate the motives of his conduct, and at the fame time learn *how little* Vaccine Inoculation has been effentially promoted by *his* endeavours. Foiled in his attempts before the Committee of the Houfe of Commons to depreciate the merits of Dr. Jenner, one would have imagined that Dr. Pearfon would defift; but in his card to you, Sir, we again find him renewing his attack, but but on different grounds; for, unmindful of his own exertions to forward the Jennerian Inoculation, he now queftions the competence of a Committee of the Britifh Houfe of Commons to decide on a fimple fact; and, talking like a lawyer, he fays, "It was coram non judice." By this, if Dr. P. means any thing, he wifhes to infinuate, that the Report ought to have little weight with the public; that doubts ought ftill to be entertained refpecting the merits of Dr. Jenner. Dr. Pearfon has advertifed a pamphlet on this fubject; but if it contains the fame fentiments as thofe delivered in his Evidence, and the fame inconfiftency as marks the fpirit of his Card to you, I will venture to predict, that its publication will only tend to increafe that difcredit which his recent conduct has attached to his name. I fhall conclude with obferving, that however great might have been the benefit which Dr. Jenner by his difcovery had conferred on fociety, if, at the fame time, it had been accompanied in him with unbounded vanity, I fhould not have been forward to vindicate his fame, and others might have rejoiced at every attempt to diminifh his reputation; but his unaffuming diffidence, and the little pains he has taken to raife his reputation, or fortune, by this great difcovery, elevate in my mind his character beyond praife, and render it a duty incumbent on every friend to virtue and to fcience to cherifh his reputation, and fhield him from the detraction of envy.

I fhall now, Sir, apologife for trefpaffing on your time fo long; but your love of juftice and deteftation of envy, will, I hope, induce you to pardon the length to which the preceding remarks have been extended. In a fhorter compafs it would have been impoffible to convey fufficiently my fenfe of the injuftice which has been done to Dr. Jenner; and fome points even, as you will readily perceive, might have admitted of more ample difcuffion than I have ventured to give them: Enough, however, has, I hope, been faid to point out *Dr. Jenner* as the man entitled to public veneration. Of his efforts I would finally fpeak in the language of Darwin's friend,

> The work is done! Nor Folly's active rage,
> Nor Envy's felf, fhall blot the golden page.

I am, &c.

C. *

*Bath, Auguft* 12, 1802.

* The intelligence difplayed in the above article, as well as our knowledge of its refpectable author, have induced us to give it a place, although we do not feel ftrongly difpofed to enter upon a controverfy, refpecting the points of which, the opinions of our well-informed readers are undoubtedly decided.

EDITORS.

